# Efficacy of *Bacillus subtilis* to replace in-feed antibiotics of broiler chickens under necrotic enteritis-challenged experiments: a systematic review and meta-analysis

**DOI:** 10.1016/j.psj.2023.102923

**Published:** 2023-07-06

**Authors:** Niati Ningsih, Adib Norma Respati, Dian Astuti, T. Triswanto, Lailatul Purnamayanti, Aan Andri Yano, Reza Pratama Putra, Anuraga Jayanegara, Adi Ratriyanto, Agung Irawan

**Affiliations:** ⁎ Department of Animal Science, Politeknik Negeri Jember, Jember 68101, Indonesia; † Agrotechnology Innovation Center, Universitas Gadjah Mada, Sleman 55573, Indonesia; ‡ Department of Feed Technology, PT. Charoen Pokphand Indonesia, Jakarta Utara 14350, Indonesia; § Animal Husbandry Study Program, Politeknik Selaparang Lombok, West Nusa Tenggara 83653, Indonesia; # Universitas Sebelas Maret, Surakarta 57126, Indonesia; ǁǁ Animal Health Vocational Program, Jambi University, Muaro Jambi 36361, Indonesia; ¶ Department of Nutrition and Feed Technology, Faculty of Animal Science, IPB University, Bogor 16680, Indonesia; ⁎⁎ Department of Animal and Rangeland Sciences, Oregon State University, Corvallis, OR 97331, USA

**Keywords:** antimicrobial resistance, alternative antibiotics, *Clostridium perfringens*, *Eimeria*, probiotics

## Abstract

Necrotic enteritis (**NE**) and coccidiosis are among the most prevalent infectious diseases in broiler chickens, contributing to large profitability losses. *Bacillus subtilis* is a promising direct-fed probiotic to counter various pathogens infection in broiler chickens. Here, we performed a meta-analysis to investigate the effects of *B. subtilis* on broiler chickens performance. A total of 28 studies were selected according to a PRISMA checklist. Random-effect model and mixed-effect model of meta-analysis were fitted to estimate the overall effects of *B. subtilis* (**BS**) treatment compared to either the control group (**CON**) or NE-infected group (**NE_inf_**) as a baseline. Hedges' *g* effect size and its variance were used as estimators of standardized mean difference (**SMD**) calculation where the results were presented at a 95% confidence interval (**95% CI**) of the SMD. Overall, NE_inf_ broiler chickens depressed (*P* < 0.01) body weight (**BW**), average daily gain (**ADG**), and feed intake, and elevated (*P* < 0.01) feed conversion ratio (**FCR**). Treatment with BS improved ADG and final BW of NE_inf_ with no difference (*P* = 0.15) between BS and antibiotics (**AB**), indicating that they had comparable efficacy to treat NE in broiler chickens. BS supplemented to uninfected CON (**BSS**) improved (*P* < 0.01) final BW, ADG, and FCR. Compared to CON, BS, and AB failed to recover the FCR but these treatments decreased (*P* < 0.01) FCR when compared to the NE_inf_ group with similar efficacy (*P* = 0.97). As expected, NE_inf_ birds had a higher mortality rate (*P* < 0.01) and higher lesion score (*P* < 0.01) compared to CON, and treatment using AB and BS successfully decreased (*P* < 0.01) the mortality rate and lesion score. Compared to BS, AB was more effective to lower (*P* = 0.01) mortality rate, but comparable (*P* = 0.65) to minimize lesion score. To conclude, *B. subtilis* could be an effective natural additive to replace in-feed antibiotics in broiler chickens challenged with *C. perfringens*. However, the efficacy to reduce mortality rate was better with antibiotics treatment.

## INTRODUCTION

Necrotic enteritis (**NE**) and coccidiosis-induced NE are among the top enteric infectious diseases in broiler chickens. Worldwide, the prevalence of these diseases is responsible for more than $6.0 billion in economic loss in the poultry industry due to sudden increases in mortality, growth impairment, high feed conversion ratio (**FCR**), as well as veterinary costs (Wade and Keyburn, 2015). NE in broilers is caused by either a direct *Clostridium perfringens* infection or coccidiosis-predisposed infection by *Eimeria* species; those species are widely presented in the poultry environment and thus can easily infect broilers if no preventive action is implemented in the farm. The high prevalence of NE and coccidiosis in broiler chickens (Alizadeh et al., 2021; Kaldhusdal et al., 2021; Aktar et al., 2022) underscores the importance of effective disease management strategies to reduce the impact on animal health and production.

Subtherapeutic in-feed antibiotics have been routinely used as a management practice to control NE and coccidiosis. However, records of antimicrobial resistance (**AMR**) in poultry are increasing due to the excessive and imprudent use of antibiotics, although laws governing more restrictive use of antibiotics have been implemented in many countries (Agunos et al., 2019; Jang et al., 2020; Romero-Barrios et al., 2020; Ali and Islam, 2021; Haider et al., 2022; Yadav et al., 2022). As AMR has become a global public health concern nowadays, establishing efficient, healthy, and sustainable preventive and protective management is critical in broiler farms to prevent AMR while optimizing profitability.

*Bacillus subtilis* is among the most popular probiotics in broiler chickens due to its wide beneficial impacts (Irawan et al., 2022; Suprayogi et al., 2022), from molecular cell improvement standpoint to growth and health enhancers benefits. In the last decade, there have been advanced-progresses in the use of *Bacillus* species in broiler chickens (El-Hack et al., 2020; Yaqoob et al., 2022), not only as a growth enhancer but also as a disease prevention (Li et al., 2017; Hayashi et al., 2018; Whelan et al., 2019). *B. subtilis*’ spore is known to be highly resistant to extreme conditions and is more stable in high temperatures of manufacture processing and storage compared to other probiotics species (Ramlucken et al., 2020a), making it a more suitable and promising feed additive in addressing the prompt of antibiotics replacement. Additionally, a growing number of studies have demonstrated that *B. subtilis* enhanced intestinal barrier integrity, mucosal immunity, digestive enzymes activities, and nutrient metabolism by mainly regulating microbial gut signatures (Zhang et al., 2021b; Yaqoob et al., 2022; Zou et al., 2022).

In broiler gut, *C. perfringens* can overgrow and elicit clinical signs of NE when predisposed to certain environmental conditions that modify gut microflora that is favorable to *C. perfringens* growth (Abd El-Hack et al., 2022). In such a challenge, *B. subtilis* helps to increase the secretion of specific antigens especially sIgA as the first line of mucosal immune defense. *B. subtilis* also secrets numerous antibacterial metabolites that could competitively reduce pathogenic and opportunistic bacteria (Sun et al., 2020; Kan et al., 2021; Oladokun et al., 2021). Several experiments have reported the capacity of *B. subtilis* to reduce the risk of bacterial disease exposure, remedy the severity of NE and coccidiosis, and reduce mortality rate in broiler chickens (Fazelnia et al., 2021; Kan et al., 2021; Liu et al., 2021). However, its protective effect on NE and coccidiosis and its recovery effect on performance have not been comprehensively studied. The efficacy among studies, additionally, varied depending on experimental conditions, dosage, age or rearing phases of broilers, sex, countries or origins, environment, and other factors. For instance, several *C. perfringens*-challenged studies reported similar efficacy between antibiotics and *Bacillus* treatments to remedy NE and coccidiosis in broiler chickens (Park et al., 2020; Kan et al., 2021) while other studies reported a higher efficacy of *B. subtilis* (Abudabos et al., 2017; Liu et al., 2021) compared to antibiotics.

Identification of various moderating variables that contribute to the discrepancies of findings among studies as outlined above is required (Sofyan et al., 2022) to better understand the efficacy of *B. subtilis* against NE and coccidiosis infections. We hypothesize that *B. subtilis* is effective and comparable with commercial antibiotics either as growth promoters or as a preventive treatment of NE in broiler chickens but variables such as the rearing periods and microbial-challenged method might contribute to the different degree of efficacy to remedy NE infection. Here, we performed a meta-analysis to provide quantitative evidence of the impacts of *B. subtilis* supplementation on performance, mortality rate, and lesion score of broiler chickens challenged with NE-induced pathogens.

## MATERIALS AND METHODS

### Search of Literature

The preferred reporting items for systematic reviews and meta-analyses (**PRISMA**) were used as a guideline for this study (Page et al., 2021). Studies that investigated the effects of *B. subtilis* in broiler chickens were searched studies from online databases including Scopus, Web of Science, and PubMed. The queries input in each database were “broiler chicken,” in combination with one of the following: “*Bacillus subtilis*,” “*Clostridium perfringens*,” “*Eimeria*,” and “necrotic enteritis.” All titles generated from each search were imported and duplicated titles were removed before further selection to identify the eligible studies. The search was conducted during February 2023 without limiting publication years.

### Inclusion Criteria

The following inclusion criteria were applied: a) peer-reviewed articles of randomized control experiments that investigated the effects of *B. subtilis* as a feed additive in broiler chickens, either in a challenged study or not; b) studies had to be ethically approved; c) included a comparison of control and intervention groups in the diets; d) reported growth performance as the main target variable; and e) provided sufficient information about the methodological details including study design, randomization, replication, intervention, data acquisition and measurement, and data analysis. Studies that were conducted under in vitro models, cross-sectional and other designs were disregarded. Studies lacking reports on growth performance parameters such as body weight (**BW**) or weight gain, feed intake (**FI**), or feed conversion ratio (**FCR**) were also excluded. Proceedings, conference abstracts, and preprints were not included in the database.

### Study Selection and Data Extraction

**Figure 1:**
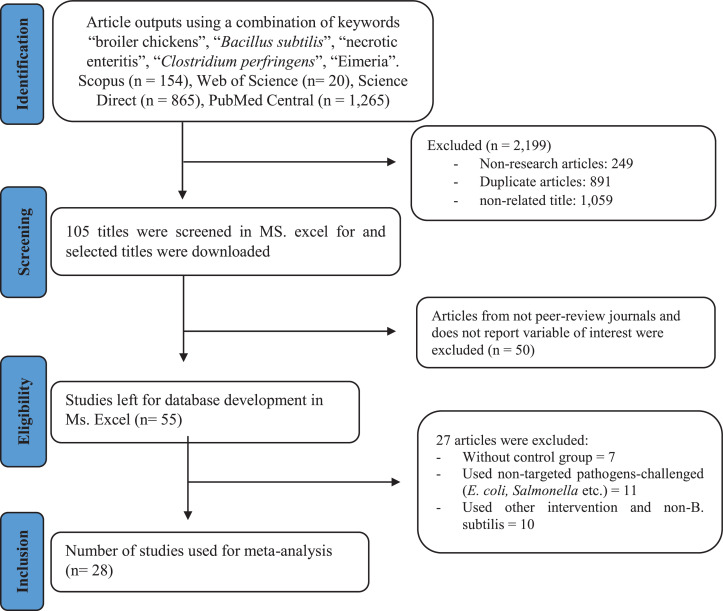
Flowchart of article selection based on PRISMA protocol.

Figure 1 represents the selection process of studies included in this meta-analysis. Pooled titles of studies retained after duplicate removal were screened by 3 researchers to determine eligibility. Selected titles from the screening were downloaded and imported to a Mendeley reference manager for further selection process by examining the full paper contents. A total of 28 studies met the eligibility criteria and relevant information was extracted into a dataset. The information includes publication details (authors, year, journal, country), the strain of broilers, number of broilers per treatment group and replicate, sex of the birds, rearing period, length of the experiment, intervention (treatment group, class or name of antibiotics when used, pathogen challenged time and method), information of diets, and variables of interests (FI, FCR, BW, ADG, mortality, lesion score) with their respective variances (standard of deviation [**SD**] or standard error [**SE**]). The SE was used in the final dataset whereas it was calculated from SD as SE = SD/sqrt(*n*); where *n* = sample size or the number of replicates. Graphical data were extracted by employing an online tool of WebPlotDigitizer (https://apps.automeris.io/wpd/) to obtain as many as possible data points (Irawan et al., 2022; Sofyan et al., 2022). A summary of the characteristics of the studies is provided in Table 1.

**Table 1:** List of studies.

No	Study	Country	Birds' strain	Sex	*N* birds	Pathogens	*Bacillus* strain	Company
1	Wang et al. (2022)	China	Arbor Acres	Mixed	200	n.a.	*B. subtilis*	n.a.
2	Hadieva et al. (2021)	Russia	Cobb	Mixed	180	n.a.	*B. subtilis* GM5	Kazan Federal University
3	Keerqin et al. (2021)	Australia	Ross 308	Male	312	*C. perfringens*	*B. subtilis* DSM 29784	Adisseo (France)
4	Liu et al. (2021)	China	Arbor Acres	Male	450	n.a.	*B. subtilis*	Kemin China, Technologies Co., Ltd., Zhuhai, China
5	Oladokun et al. (2021)	Canada	Cobb 500	n.a.	450	n.a.	*B. subtilis*	Probiotech International, St. Hyacinth, QC, Canada
6	Poudel et al. (2021)	USA	Ross 708	Male	1248	*Eimeria* sp.	*B. subtilis* PB6	(CLOSTATR Dry, Kemin Indus- tries, Iowa)
7	Wang et al. (2021b)	China	Lingnan	Mixed	360	*C. perfringens*	*B. subtilis* DSM29784 (BS)	Chinese Academy of Sciences (Beijing, China).
8	Wickramasuriya et al. (2021)	USA	Ross 308	n.a.	80	*E. acervulina*	*B. subtilis*-EV	
9	Xu et al. (2021)	China	Cobb 500	Male	360	n.a.	*B. subtilis* (HJKC02) and *B. licheniformis* (HJDY01)	Vegamax Biotechnology Co., Ltd. (Zhejiang, China).
10	Yang et al. (2021)	China	Cobb	Mixed	500	n.a.	*B. subtilis* yb-114,246	Anhui Academy of Agricultural Sciences
11	Zeng et al. (2021)	China	Arbor Acres	Mixed	1200	n.a.	*C. butyricum*-, *B. subtilis*-, and *B. licheniformis*	n.a.
12	Zhang et al. (2021b)	China	Arbor Acres	Male	270	n.a.	*B. subtilis* (LIFEGUFS-S 200)	Lifecome Biochemistry Co., Ltd.
13	Bilal et al. (2021)	Canada	Cobb	Male	2073	n.a.	*B. subtilis* and *B. pumilus*	Lallemand SAS, Blagnac, France
14	Park et al. (2020)	USA	Ross 708	Male	196	*Eimeria maxima*	*B. subtilis* 1781 and 747	Church & Dwight Co., Inc. (Waukesha)
15	Ramlucken et al. (2020b)	South Africa	Ross 308	Male	1656	*C. perfringens Type A*	*B. subtilis* (CPB 011, CPB 029, HP 1.6, and D 014) and *B. velezensis* (CBP 020 and CPB 035)	CSIR (Biosciences, Pretoria, South Africa)
16	Wang et al. (2021a)	China	Lingnan	Mixed	1200	n.a.	*B. subtilis*	
17	Aljumaah et al. (2020)	Saudi Arabia	Ross 308	Mixed	100	*C. perfringens*	*B. subtilis* PB6	CloStat, Kemin Industries
18	Bortoluzzi et al. (2019)	USA	Cobb	Male	1200	*C. Perfringens*	*B. subtilis* DSM 32315	n.a.
19	Whelan et al. (2019)	Germany	Ross 308	Male	462	*C. perfringens + Eimeria maxima*	*B. subtilis* DSM 32315	n.a.
20	Sokale et al. (2019)	USA	Cobb	Male	320	*C. perfringens + Eimeria maxima*	*B. subtilis* DSM 32315	n.a.
21	Wang et al. (2019)	China	Ross 308	Male	1440	*C. perfringens* and *Eimeria* sp.	*B. subtilis* strains; 2084, LSSAOl, and 15A-P4	n.a.
22	Cheng et al. (2018)	Taiwan	Ross 308	Female	60	*C. perfringens*	*B. subtilis* NIU068	Chiao-Xi hot spring in Taiwan
23	Wang et al. (2018)	USA	Ross × Ross 708	Male	1344	*Eimeria oocysts*	*B. subtilis* PB6	n.a.
24	Abudabos et al. (2017)	USA	Ross 308	Mixed	480	*C. perfringens*	*B. subtilis* (ATCC PTA-6737)	Clostat, Kemin Industries Inc., Des Moines, IA
25	Koli et al. (2018)	India	Vencobb- 400	Mixed	400	*C. perfringens*	*B. subtilis* (GalliPro-DSM 17299)	n.a.
26	Abudabos et al. (2013)	Saudi Arabia	Ross 308	Male	100	*C. perfringens*	*B. subtilis* PB6 (CloSTAT)	Kemin Industries Inc., Des Moines, IA
27	Jayaraman et al. (2013)	India	Cobb 400	Mixed	216	*Eimeria* sp. and *C. perfringens*	*B. subtilis* PB6 (ATCC-PTA 6737)	Kemin Industries South Asia Private Limited manufacturing facility, Gummidipoondi, India
28	Tactacan et al. (2013)	Georgia	Cobb	Male	400	*C. perfringens*	*B. subtilis* (QST 713)	n.a.

### Study Assessment

**Figure 2:**
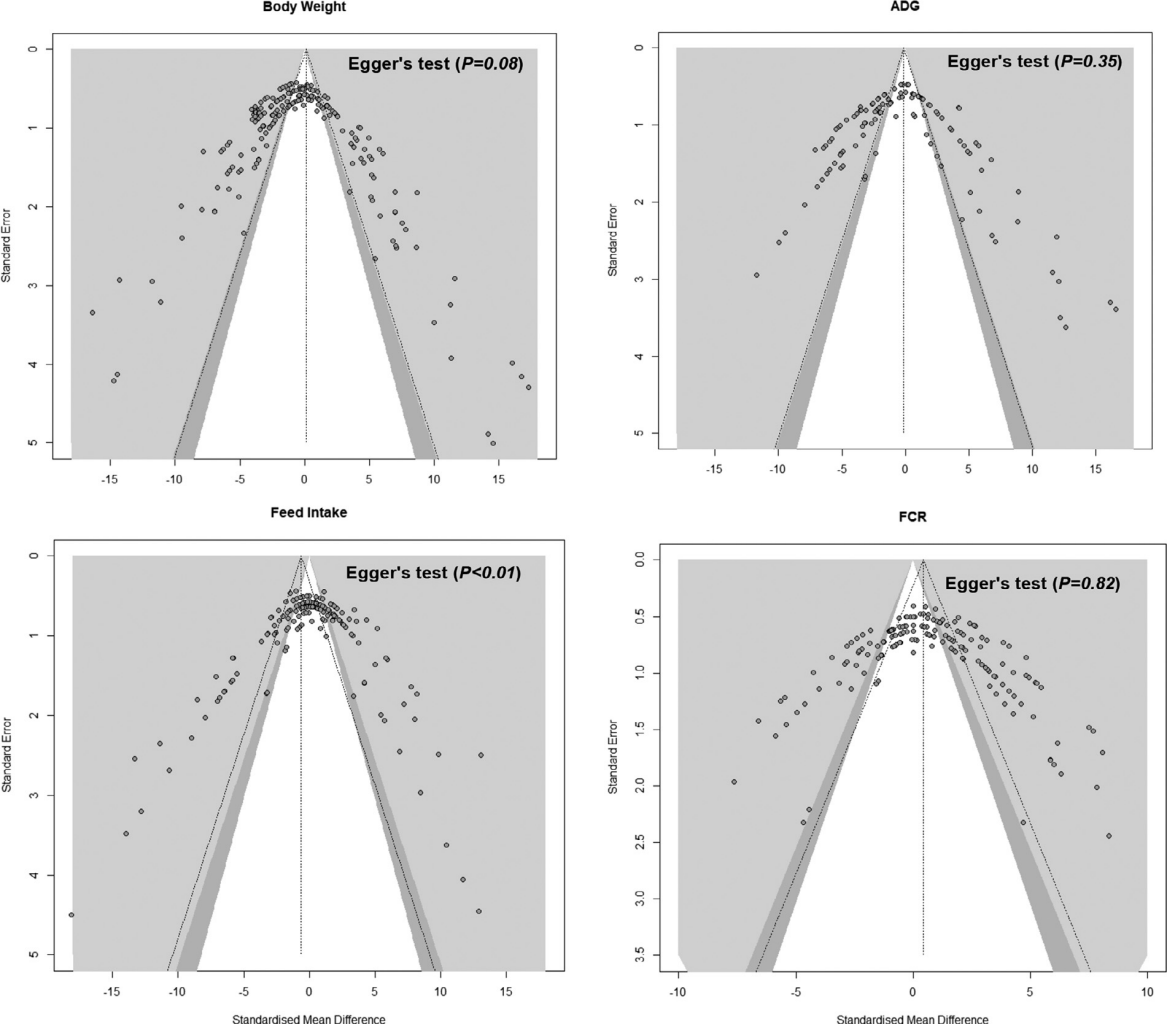
Traffic light plots and weighted bar plots represent the results of risks of bias assessment studies included in the meta-analysis (green means low risk of bias, yellow means unclear risk of bias, red means high risk of bias).

We adopted the Cochrane quality assessment method to assess the risk of bias in studies included in this meta-analysis. For each study, the following 6 indicators were assessed: i) bias arising from the randomization process, ii) bias due to reporting procedure/method, iii) bias due to statistical approach, iv) bias due to deviations from intended intervention, v) bias due to the missing outcome and outcome's variance, and vi) bias in the selection of the reported result. Each item was given a score of 0 for “high risk,” 1 for “low risk,” and 2 for “no risk” of bias. Three researchers were involved in the risk of bias assessment and the total score was computed. Studies with a total score ≤18 or having a risk for all indicators were excluded. The traffic light and weighted bar plots representing the results of risks of bias assessment are provided in Figure 2.

### Sensitivity Analysis and Publication Bias Measurement

**Figure 3:**
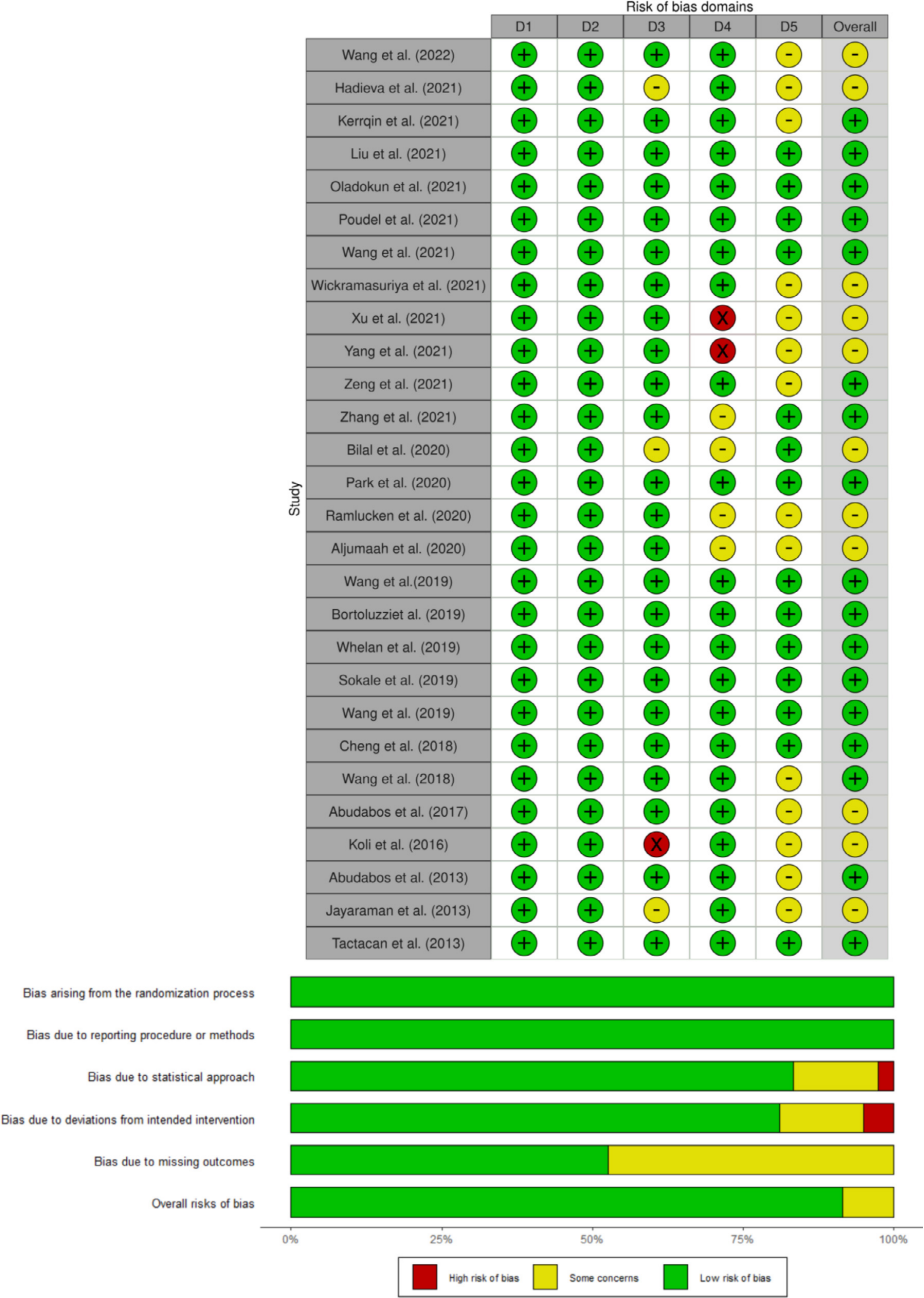
Funnel plots to assess the publication bias of the studies. Egger's test results were included in each outcome of the plot.

In addition to quality assessment, sensitivity analysis was conducted to identify the stability of the treatment effects by examining the influential studies that have a high impact or contribute to high heterogeneity on the variables measured. This was done using a leave-one-out analysis (Viechtbauer, 2010). It was expected that high heterogeneity was present by nature in the typical in vivo experiment of broilers especially in a pathogens-challenged experiment. No study was removed from the dataset based on the sensitivity analysis, indicating that all data points were robust. Publication bias was examined by using funnel plots (Figure 3) and Egger's test (Egger et al., 1997) and is considered significant for *P* ≤ 0.05. Publication bias evidence was observed for feed intake and mortality (*P* < 0.05), likely due to large across-studies heterogeneity, especially for mortality. However, no extreme value was observed as visualized in the funnel plots as well as according to the leave-one-out sensitivity analysis. Therefore, no study or data points were removed at this step.

### Statistical Analysis

In most studies, multiple comparisons were tested over the control group (**CON**), including i) CON challenged with NE (**NE_inf_**), ii) NE_inf_ treated with *B. subtilis* (**BS**), iii) NE_inf_ treated with antibiotics (**AB**), iv) CON supplemented with *B. subtilis* as probiotics supplement (**BSS**), and v) CON plus antibiotics as a growth promoter (**ABS**). Therefore, a subgroup meta-analysis was applied to compare the efficacy of *B. subtilis* either as a treatment model NE_inf_ studies or as a growth promoter model in comparison to antibiotics.

All analyses were performed in the R studio environment (RStudio version 4.1.1) and meta-analysis was conducted using the “meta” package (Schwarzer et al., 2015). Between-study heterogeneity of each response variable was assessed using Cochran's *Q* statistic and *I*^2^ statistic (Higgins et al., 2003). The DerSimonian-Laird estimator was used to estimate the *I*^2^ statistic which explains the total variance (%) across studies. The *I*^2^ was classified as high (*I*^2^ ≥ 75%), moderate (50% < *I*^2^ ≤ 75%), low (25% < *I*^2^ ≤ 50%), and with no evidence of heterogeneity (0 < *I*^2^ ≤ 25%). The results of the heterogeneity test indicated that all variables of interest had a high heterogeneity (*I*^2^ ≥ 75%). Therefore, random-effect model (**REM**) analysis was fitted to estimate the overall effects of the study interventions vs. control on the variable outcomes. By default, the Hedges' *g* effect size and its variance were used to estimate the standardized mean difference (**SMD**), weighted by the inverse-variance matrix from the studies using the following equations:

(1) $$\text{SMD}=\;\frac{\mu_{1}-\;\mu_{2}}{\text{SP}}\text{SP}$$

(2) $$\text{where}\;\text{SP}\;=\;\sqrt{\frac{\left(n_{1}-1\right){\text{SD}}_{1}^{2}+\left(n_{2}-1\right){\text{SD}}_{2}^{2}}{n_{1}+n_{1}-2}}$$

(3) $$\text{and}\;\text{inverse}-\text{variance}\;\text{weighted}\;=\;\frac{\sum Yi\left(1/{\text{SE}}_{i}^{2}\right)}{\sum \left(\frac{1}{{\text{SE}}_{i}^{2}}\right)}$$

where *μ*_1_ is the mean of the dietary intervention group, *μ*_2_ is the mean of the control group, SP is the combined SD, SD_1_ is the SD of the dietary intervention group, SD_2_ is the SD of the control group, *n*_1_ is the sample size of the dietary intervention group, *n*_2_ is the sample size of the control group, *Yi* is the estimates of the intervention effect in *i* study, SE*_i_* is the SE of the estimate (Schwarzer et al., 2015). The SMD was presented as a 95% confidence interval (**95% CI**) and the results were illustrated in forest plots.

Furthermore, a mixed-effects model was fitted to examine the interaction effects of moderating variables with dietary interventions that might interfere with the outcomes (Irawan et al., 2022; Rusli et al., 2022). This includes rearing phases (starter, grower, finisher), the sex of the birds identified in the article (male, female, mixed), country of origin, and strain of broilers. All variable outcomes included in the analysis were from ≥5 different studies which are considered to have sufficient statistical power to obtain robust results (Irawan et al., 2022; Rusli et al., 2022).

## RESULTS

### Study Characteristics

The search of literature resulted in 1,261 titles from PubMed Central, Scopus, Web of Science, and Science Direct. After eliminating the overlapped or duplicated titles, 691 titles were retained and were checked for more details, resulting in 105 studies left. Of these, 50 studies were excluded due to nonrelevant abstract content. Finally, the full text of these remaining titles was downloaded and examined thoroughly, resulting in 28 final studies that were eligible to be included in this meta-analysis.

The selected studies were all randomized control trials, published between 2013 and 2022 where 21 of them were published between 2019 and 2022 (Table 1), indicating that interest in the use of *B. subtilis* is growing especially to remedy the prevalence of NE and coccidiosis in broiler chickens. The studies were most frequently conducted in China (9/28) and the USA (7/28) while studies in other countries including Australia, Canada, Georgia, Germany, India, Russia, Saudi Arabia, South Africa, and Taiwan were less frequent (1–2 studies). The representation of the country of origin across studies indicated that NE and coccidiosis in broiler chickens are global problems for the broiler industry. Strains of broilers used in the studies were quite homogenous whereas Ross and Cobb accounted for 75% of predominant strains while the rest of the studies used Arbor Acres, Vencobb, and Lingnan. The number of birds in each study was highly variable from 60 to 2,073 birds, ≥50% were male broilers. Of all included studies, NE-induced conditions were performed in 18 studies while the other 10 studies examined the effect of *B. subtilis* as a growth enhancer without NE-challenged. The NE induction methods were performed either using *C. perfringens* (10 studies), *Eimeria*-induced NE (4 studies) or used a combination of *C. perfringens* and *Eimeria* (4 studies) as a model to induce NE disease in broiler chickens. The dataset showed that *B. subtilis* mostly produced by commercial companies such as Kemin Industries Inc. (the most frequently used), Adisseo, Lallemand, Lifecome Biochemistry Co., Ltd., Probiotech International, Vegamax Biotechnology Co., Ltd., and Church % Dwight Co., Inc., with few were produced by a laboratory in universities (4 studies). Overall, the characteristics of the studies indicate that the data are robust and trustworthy.

### Meta-Analysis on Performance Parameters

Examination of moderating variables contribute to the outcome variables is presented in Table 2. Significant effects of dietary treatments were observed on BW, ADG, FI, FCR, and mortality (*P* < 0.01); this justified the suitability of subgroup meta-analysis to compare the treatment groups vs. CON. To further examine the efficacy between *B. subtilis* and antibiotics on NE_inf_ birds, another subgroup meta-analysis was performed using the NE_inf_ group as a baseline.

**Table 2:** Effects of various explanatory variables to the estimated outcomes.

Outcomes	*n*	*P* value of moderating variables				
		Trt	Phase	Sex	Country	Strain
Body weight	237	<0.01	0.084	0.016	<0.001	0.194
Average daily gain	126	<0.01	0.505	0.040	<0.001	0.134
Feed intake	185	<0.01	0.002	0.010	<0.001	0.281
Feed conversion ratio	200	<0.01	0.247	0.001	<0.001	0.391
Mortality	34	<0.01	-	0.162	<0.001	-
Lesion score	28	0.62	-	0.667	<0.001	-

Abbreviation: Trt, treatments.

**Figure 4:**
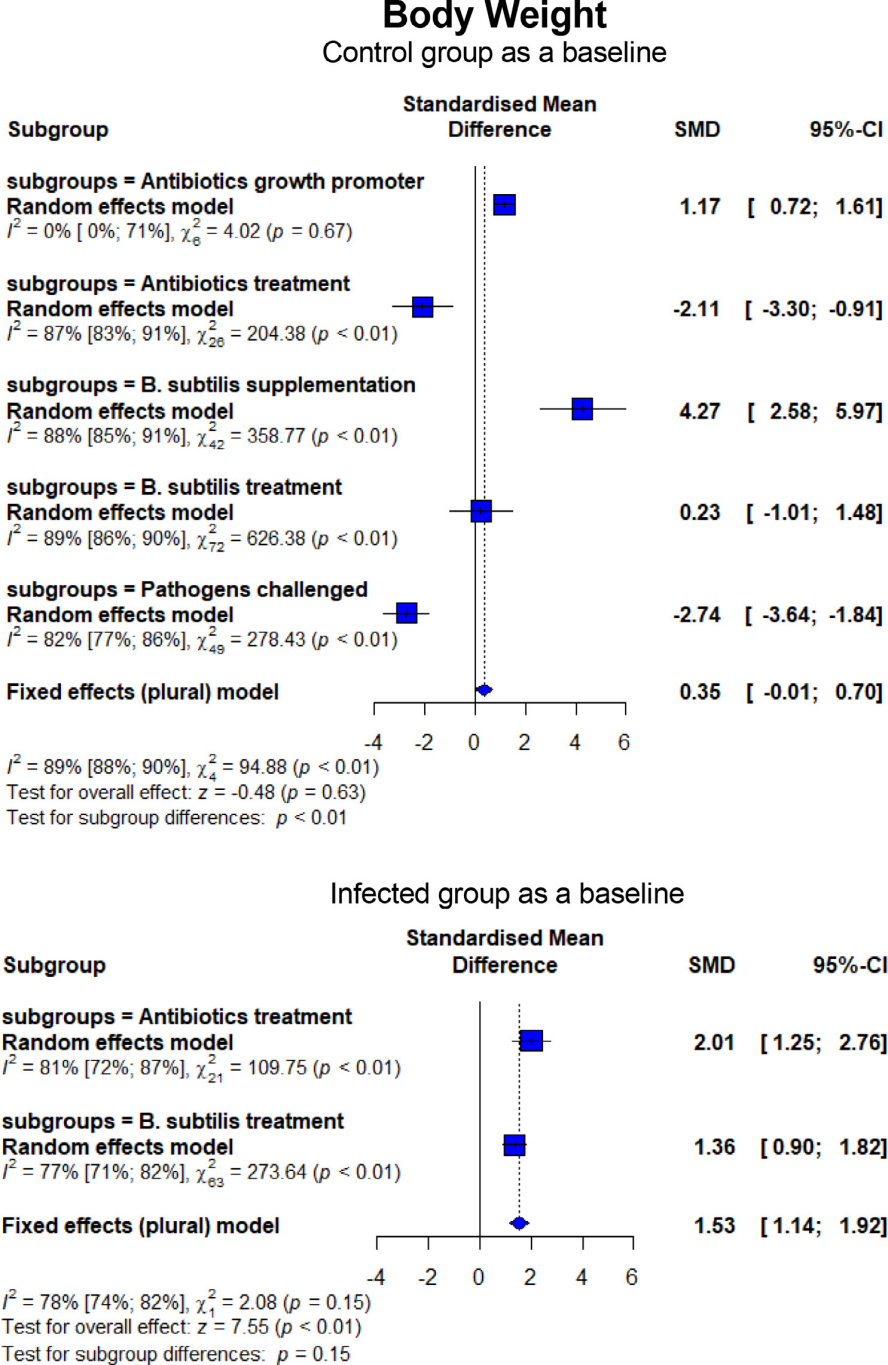
Forest plot of BW showing the 95% CI (lower–upper) of the SMD between the means of groups of dietary treatment and group of control diet. The *x*-axis shows the SMD; central-dashed line represents the zero effect (SMD = 0) of dietary interventions; blue-diamonds represent the overall effect while the specific symbols in each line represent the SMD (subgroup effect) of the specific group. Reduction effects are reflected when the SMDs are on the left of the central dashed-line and increasing effects are in opposite (to the right of the line).

**Figure 5:**
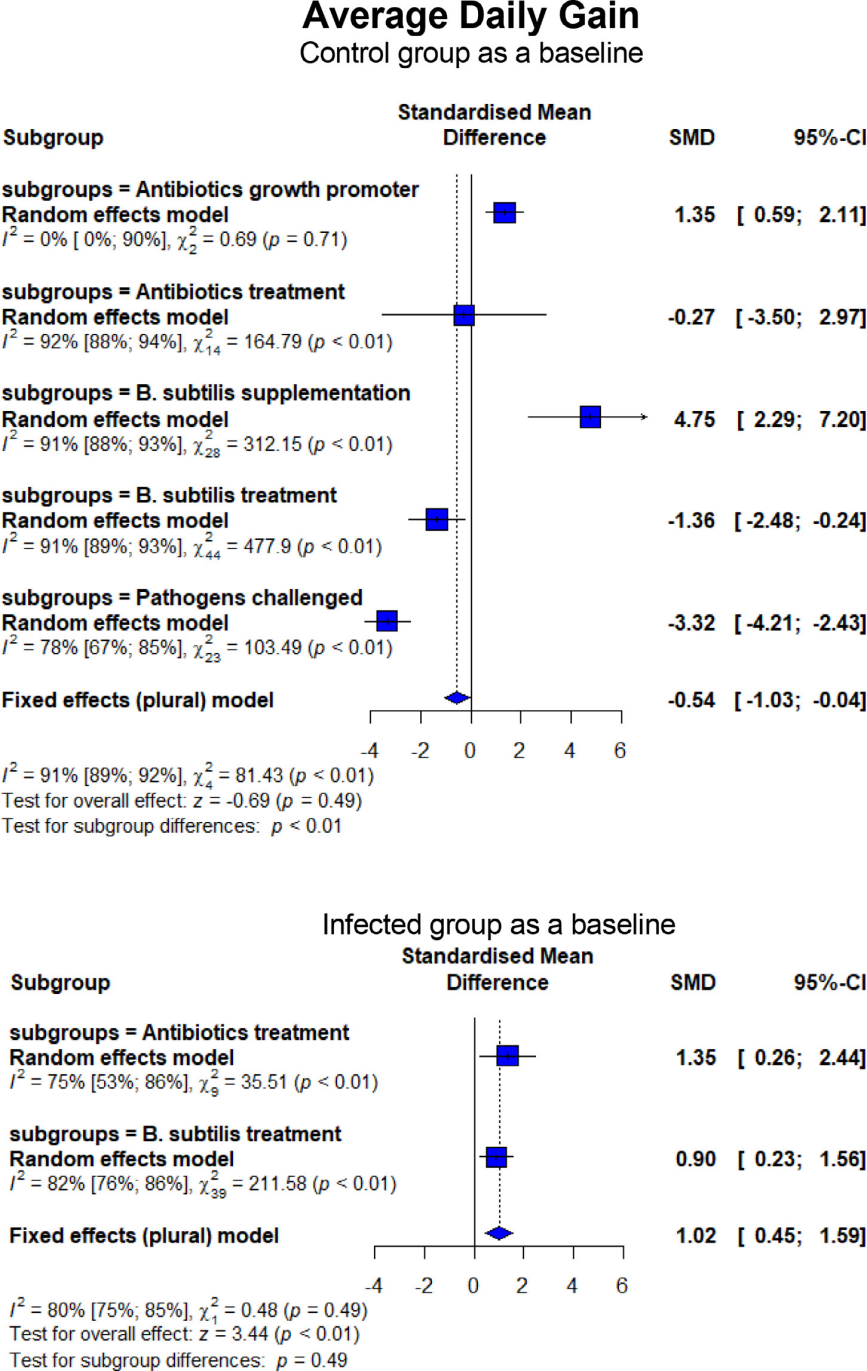
Forest plot of ADG showing the 95% CI (lower–upper) of the SMD between the means of groups of dietary treatment and group of control diet. The *x*-axis shows the SMD; central-dashed line represents the zero effect (SMD = 0) of dietary interventions; blue-diamonds represent the overall effect while the specific symbols in each line represent the SMD (subgroup effect) of the specific group. Reduction effects are reflected when the SMDs are on the left of the central dashed-line and increasing effects are in opposite (to the right of the line).

**Figure 6:**
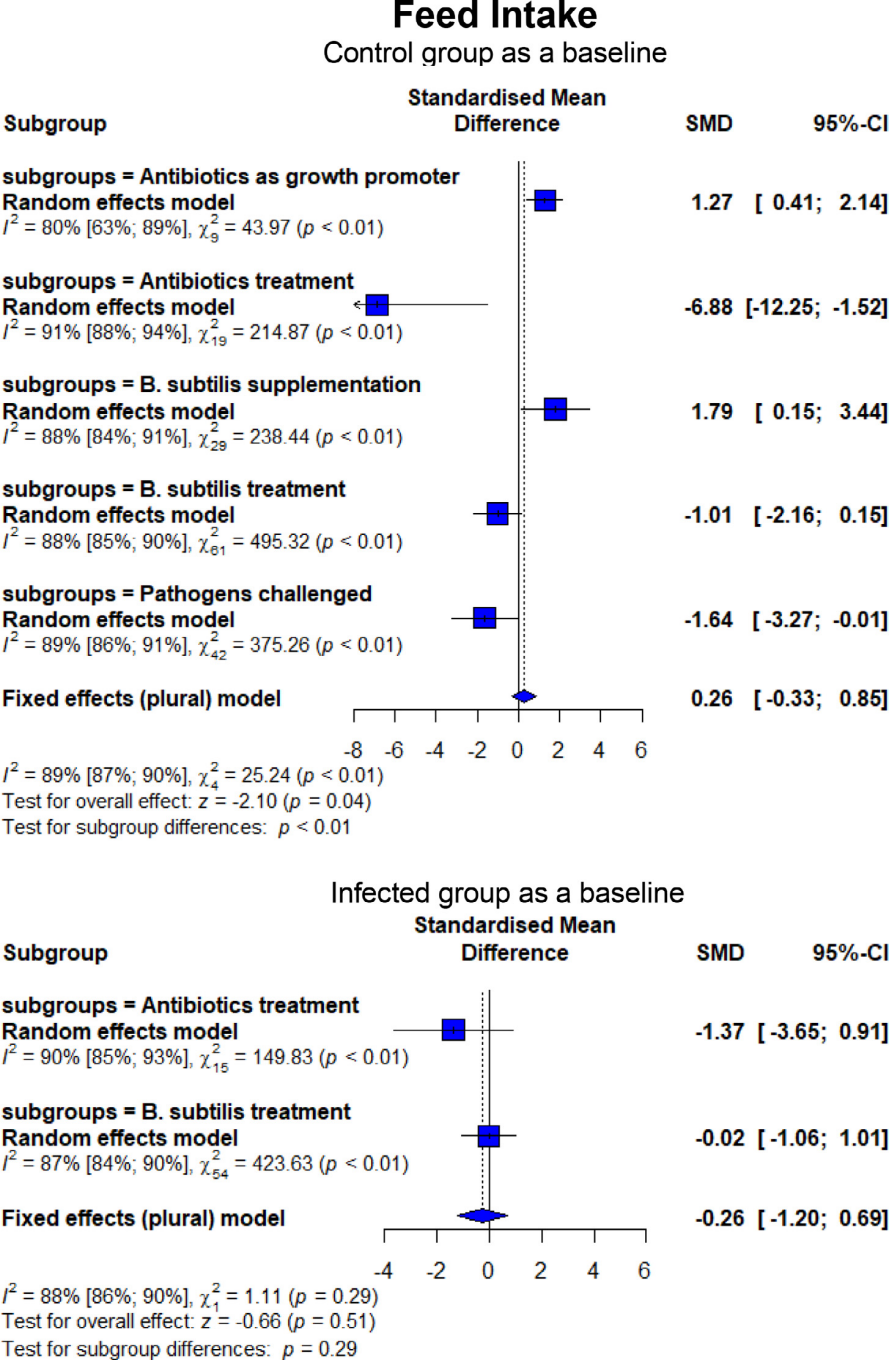
Forest plot of FI showing the 95% CI (lower–upper) of the SMD between the means of groups of dietary treatment and group of control diet. The *x*-axis shows the SMD; central-dashed line represents the zero effect (SMD = 0) of dietary interventions; blue-diamonds represent the overall effect while the specific symbols in each line represent the SMD (subgroup effect) of the specific group. Reduction effects are reflected when the SMDs are on the left of the central dashed-line and increasing effects are in opposite (to the right of the line).

**Figure 7:**
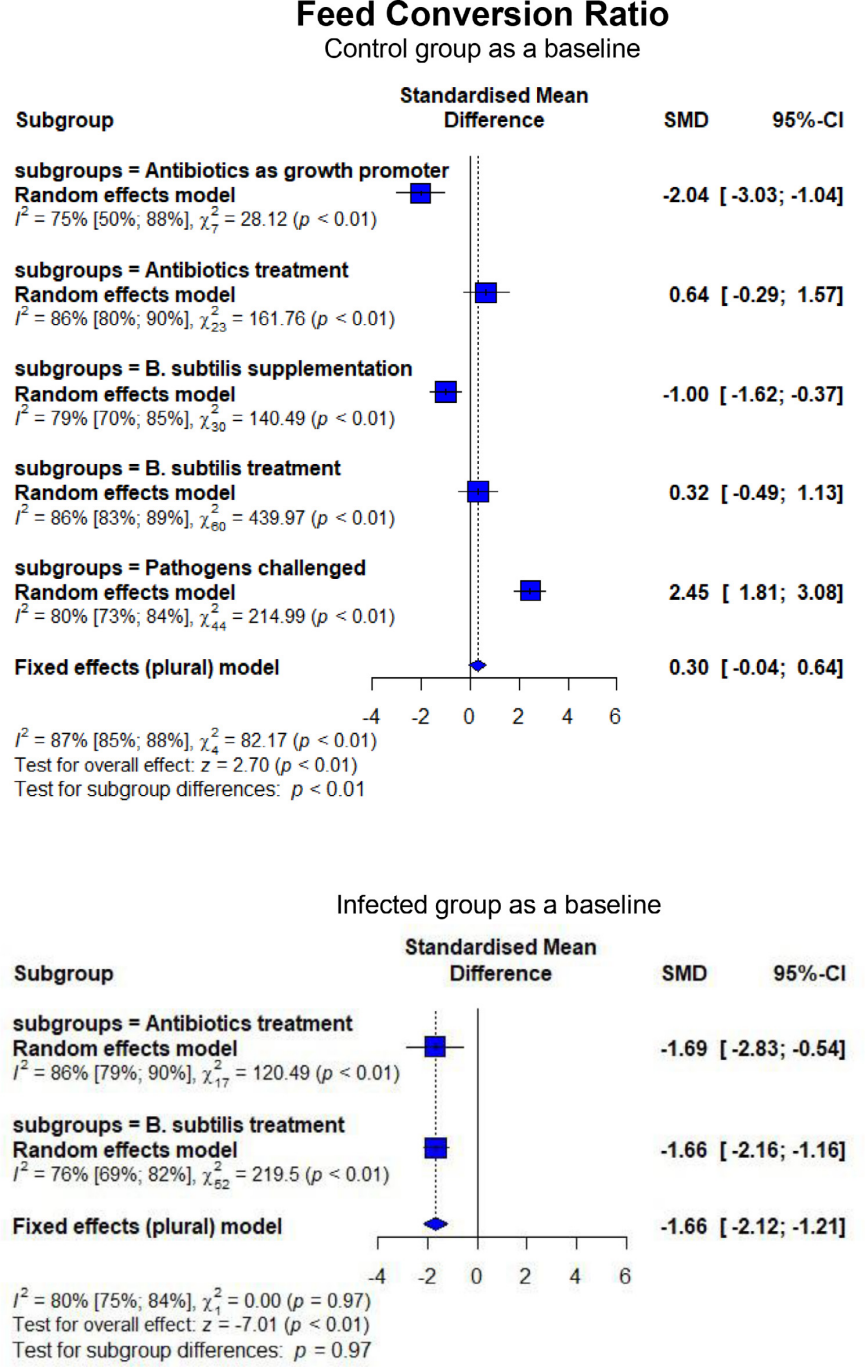
Forest plot of FCR showing the 95% CI (lower–upper) of the SMD between the means of groups of dietary treatment and group of control diet. The *x*-axis shows the SMD; central-dashed line represents the zero effect (SMD = 0) of dietary interventions; blue-diamonds represent the overall effect while the specific symbols in each line represent the SMD (subgroup effect) of the specific group. Reduction effects are reflected when the SMDs are on the left of the central dashed-line and increasing effects are in opposite (to the right of the line).

**Figure 8:**
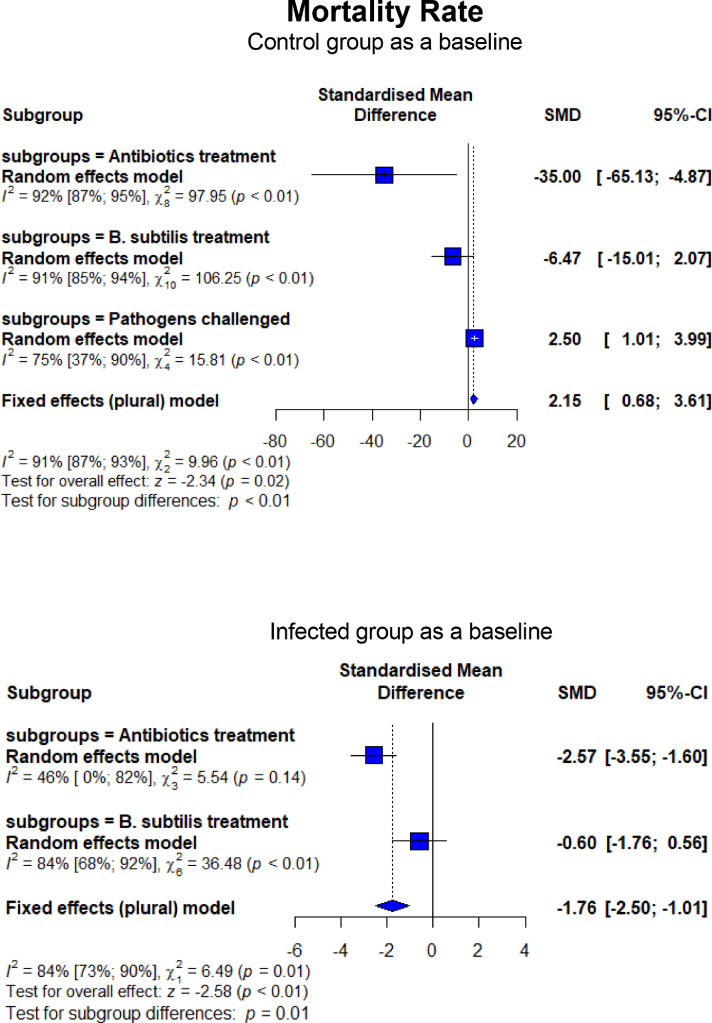
Forest plot of mortality rate showing the 95% CI (lower–upper) of the SMD between the means of groups of dietary treatment and group of control diet. The *x*-axis shows the SMD; central-dashed line represents the zero effect (SMD = 0) of dietary interventions; blue-diamonds represent the overall effect while the specific symbols in each line represent the SMD (subgroup effect) of the specific group. Reduction effects are reflected when the SMDs are on the left of the central dashed-line and increasing effects are in opposite (to the right of the line).

**Figure 9:**
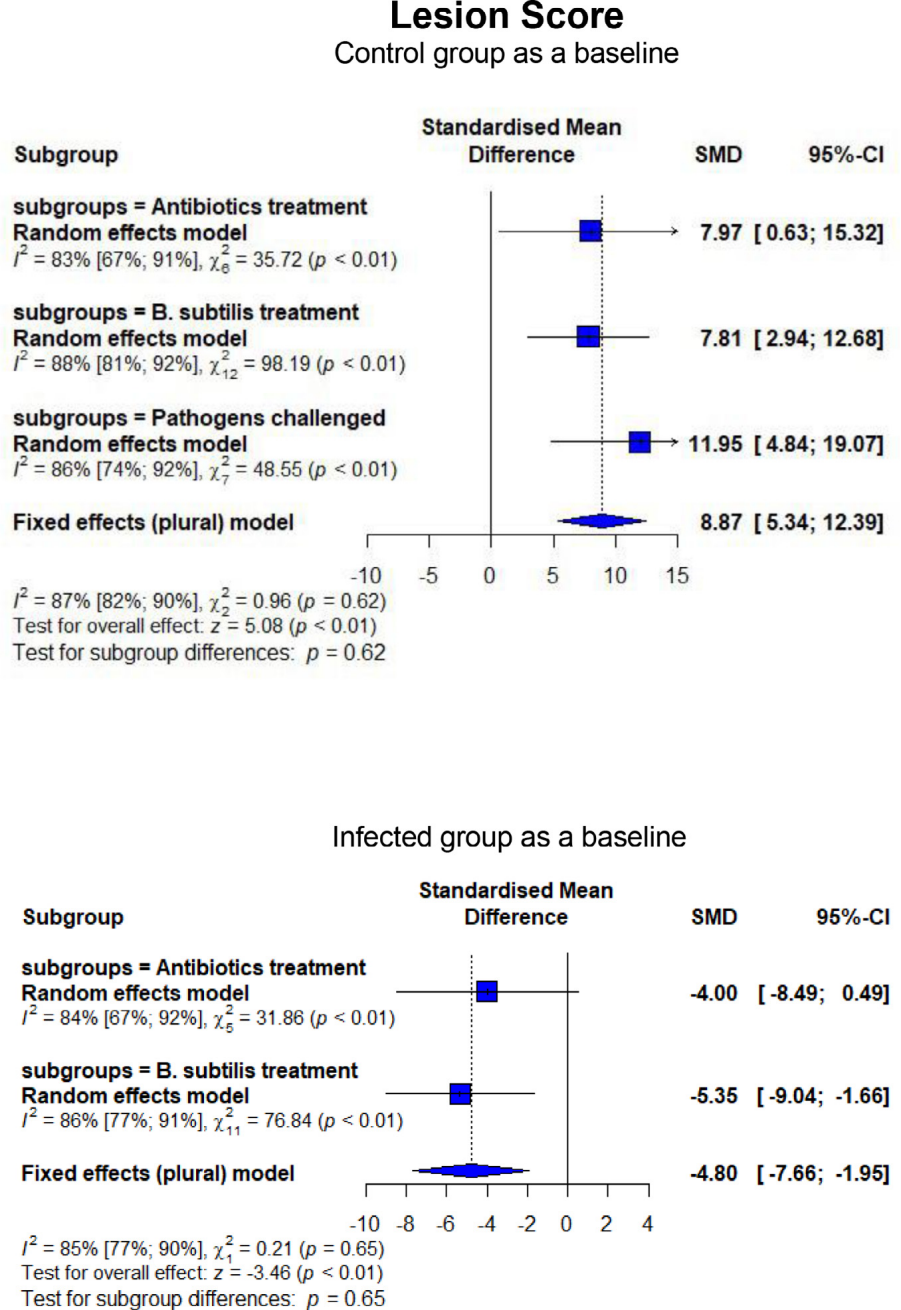
Forest plot of lesion score showing the 95% CI (lower–upper) of the SMD between the means of groups of dietary treatment and group of control diet. The *x*-axis shows the SMD; central-dashed line represents the zero effect (SMD = 0) of dietary interventions; blue-diamonds represent the overall effect while the specific symbols in each line represent the SMD (subgroup effect) of the specific group. Reduction effects are reflected when the SMDs are on the left of the central dashed-line and increasing effects are in opposite (to the right of the line).

The forest plots summarizing the results of the meta-analysis are presented in Figures 4 to 9. Overall, broiler chickens infected with *C. perfringens* or *Eimeria* that induced NE disease experienced significant depression on BW (SMD = −2.74, 95% CI = −3.64 to −1.84, *P* < 0.01) and ADG (SMD = −3.32, 95% CI = −4.21 to −2.43, *P* < 0.01). Compared to CON, dietary treatment with BS and AB had lower (*P* < 0.01) BW and ADG (Figure 4). However, BS and AB resulted in higher final BW compared to the NE_inf_ group (SMD = 2.01, 95% CI = 1.25–2.76, *P* < 0.01 for AB; SMD = 1.36, 95% CI = 0.90–1.82, *P* < 0.01 for BS, respectively). No difference (*P* = 0.15) was found between AB and BS for their improvement effect on NE_inf_, indicating that they had a comparable efficacy to treat NE in broiler chickens. Similar trends were also observed in ADG whereas AB and BS had higher (*P* < 0.01) ADG compared to the NE_inf_ group but no significant difference was found between those treatment groups (*P* = 0.49; Figure 5). When given as a probiotics supplement (BSS), it remarkably increased BW (SMD = 4.27, 95% CI = 2.58–5.97, *P* < 0.01) and ADG (SMD = 4.75, 95% CI = 2.29–7.20, *P* < 0.01) than CON. Interestingly, ABS had a lack of growth-promoting effect on BW (*P* = 0.67) and ADG (*P* = 0.71).

As shown in Figure 6, broilers experimentally infected with pathogens-induced NE had lower FI (SMD = −1.64, 95% CI = −3.27 to −0.01, *P* < 0.01) and higher FCR (SMD = 2.45, 95% CI = 1.81–3.08, *P* < 0.01). In contrast, BS treatment lowered (*P* < 0.01) FI compared to CON and NE_inf_, similar to what was observed for AB on FI (*P* = 0.01). An improvement (*P* < 0.01) in FCR was found when BS was supplemented to NE_inf_ birds, although BS failed to recover the FCR to be similar to CON. The effect of BS on FCR was equal (*P* = 0.97) to AB (Figure 7). In the CON group receiving AGP and BSS, higher FI (*P* < 0.01) FI and lower (*P* < 0.01) FCR were observed. Infection with *C. perfringens* and *Eimeria* resulted in a higher mortality rate than CON (SMD = 2.50, 95% CI = 1.01–3.99, *P* < 0.01; Figure 8). Treatment of in-feed AB and BS successfully decreased the mortality rate (SMD = −6.47, 95% CI = −15.01 to 2.07, *P* < 0.01 for BS and SMD = −35.00, 95% CI = −65.13 to −4.87, *P* < 0.01 for AB, respectively). Evidence of a more effective effect of AB vs. BS was found as shown by a significantly lower (*P* = 0.01) mortality rate for AB (SMD = −2.57, 95% CI = −3.55 to −1.60) than that of BS (SMD = −0.60, 95% CI = −1.76 to 0.56). Experimentally infected broilers also showed a higher lesion score (SMD = 11.95, 95% CI = 4.84–19.07, *P* < 0.01) compared to CON (Figure 9). Infected broilers treated with AB and BS also had higher lesion scores than the CON group, but they were effective to reduce the lesion score as shown in significantly lower (*P* < 0.01) SMD estimates compared to CH as a baseline. No difference was found between AB and BS in treating the infection (*P* = 0.65; Figure 9).

## DISCUSSION

The synthesis results of the present meta-analysis revealed that *B. subtilis* was overall efficacious to diminish the deleterious effects of *C. perfringens* and *Eimeria* infections in broiler chickens. This was shown by the higher BW and ADG of infected birds when administered with *B. subtilis*. Despite the lack of recovery effect, *B. subtilis* demonstrated similar efficacy to antibiotics. High FCR has become one of the economic loss-driving factors in commercial broiler farms with NE disease alleviation. The use of *B. subtilis* was able to recover the FCR to be not significantly different from noninfected birds. The recovery rate of FCR was also similar to antibiotics administration. NE is a multifactorial disease that can be directly induced by *C. perfringens* infection during the finisher phase of broiler chickens. In addition, coccidia parasites especially *Eimeria acervulina, E. tenella*, and *E. maxima* can also develop NE due to intestinal necrosis that can promote *C. perfringens* to grow (Abd El-Hack et al., 2022). In the intestine, various toxins are released by *C. perfringens*, leading to the development of NE disease. This condition consequently suppresses both innate and cell-mediated immunity of broiler chickens and impaired growth performance and efficiency.

*B. subtilis* possesses many substantial roles in improving gut microbial ecosystems, immunity, nutrient utilization, and maintaining the health status of broilers (Chaudhari et al., 2020; Fazelnia et al., 2021; Wang et al., 2021a; Yaqoob et al., 2022) and thus helps to minimize the adverse effects of pathogens infection. The functionality of *B. subtilis* to modulate gut commensal microbes and enhance mucosal immunity is well documented in the literature, which explains the favorable effects on performance improvement. Evidence of microbial gut modulation by *B. subtilis* in broiler chickens under the NE challenge was reported in several recent studies. For instance, Wang et al. (2021b) found an increase in the abundance of *Ruminococcaceae* and *Bifidobacterium* families in broilers’ cecum fed with *B. subtilis*. The increase of these bacterial populations explains the ability of *B. subtilis* to reduce the negative effect of NE. An increase of beneficial bacteria such as *Bacteroidetes* phylum and *Bacteroidia* class was reported by using 3 different *B. subtilis* strain to encounter pathogens infection (Wang et al., 2019). An increase in the *Bacteroidia* population has been suggested to facilitate better nutrient digestion in broilers and thus contribute to performance improvement (Thomas et al., 2011; Li et al., 2016). In the case of NE incidence, *B. subtilis* DSM 32315 was reported to be able to control the *C. perfringens* proliferation and competitively prevent the growth of *Campylobacter jejuni, Escherichia coli*, and *Salmonella* spp. (Whelan et al., 2019).

Additionally, it was previously suggested that intestinal diseases such as coccidiosis and NE are sources of stressors that trigger oxidative stress in broilers due to the interactions of their toxins and intestinal mucosa. Such negative interaction leads to excessive free radical formation that could destroy the epithelial barrier, lipid peroxidation, antioxidant insult, DNA damage, and apoptosis (Mishra and Jha, 2019). In this regard, *B. subtilis* exhibits a scavenging agent to enhance antioxidant capacity of broilers and thus can be an excellent additive to diminish redox imbalance of broilers that are exposed to stressors including disease prevalence. Several studies have shown that *B. subtilis* could positively affect key enzymes in the redox balance such as glutathione peroxidase (**GSH-Px**) and superoxide dismutase (**SOD**) which contribute to enhance overall total antioxidant capacity (**T-AOC**). Wang et al. (2021a) and Zhang et al. (2021a) reported that *B. subtilis* administration increased GSH-Px and SOD activities, increased T-AOC in the serum and the jejunum, and reduced malondialdehyde (**MDA**) concentration of the intestine of broilers. In a recent experiment, Wickramasuriya et al. (2021) demonstrated that the positive effect of *B. subtilis* on antioxidant status was indirect via the effective delivery of cNK-2 peptide, an antimicrobial peptide that has the ability to reduce oxidative stress and enhance gut integrity induced by intestinal microbial infection.

The above positive modulatory mechanism in the gut may explain the positive effects of *B. subtilis* on the health status of broiler chickens (Poudel et al., 2021; Wang et al., 2021a; Yaqoob et al., 2022). The capacity of *B. subtilis* to synthesize a plethora of antimicrobial compounds and to form a high-survival endospores in the gastrointestinal tract also supports the host microbiome balancing mechanism. The antimicrobial compounds include organic and inorganic volatile compounds, ribosomal and nonribosomal peptides, polyketides, and other various chemical compounds (Caulier et al., 2019; Ramlucken et al., 2020a; Su et al., 2020). Among thousands of identified volatile compounds produced as secondary metabolites by *B. subtilis*, volatile fatty acids such as butyric and acetic acids represent approximately 87% of compounds having antimicrobial properties (Caulier et al., 2019). An increase in butyric acid and acetic acid concentration and concomitant improvement in the performance of broiler chickens were reported in *C. perfringens*-infected birds by treating *B. subtilis* (Aljumaah et al., 2020). An increase in lactic, succinic, nicotinic, propionic, and malic acids was also reported in NE-challenged broilers administered with *B. subtilis* DSM29784 (Wang et al., 2021b). Other important compounds that are produced by *B. subtilis* are ribosomal peptides, a class of compounds that are formed from short amino acid precursors and are popularly also referred to as bacteriocins. Bacteriocins, bacteriocins-like compounds, and antimicrobial peptides (**AMP**) exhibit a wide inhibitory capacity against various pathogens. Specifically, they are involved in cell destruction via pore formation or DNA/RNA disruption leading to cell wall synthesis inhibition of pathogens (Ramlucken et al., 2020a). A cytotoxic effect of AMP produced by *Bacillus* spp. was reported to suppress the population of *Eimeria* and *C. perfringens* and subsequently reduce coccidiosis and NE prevalence in broiler chickens (Lee et al., 2010).

In a recent high throughput metabolomic-transcriptomic study, Zou et al. (2022) demonstrated that *B. subtilis* has strong roles to upregulate inflammatory response-associated genes, modulating gut microbial signature by enriching beneficial bacteria and suppressing harmful bacteria, and increase N-acetylneuraminic acid and ADP metabolites. Enrichment of those metabolites suggests an improved oxidative status of the birds as it exerts anti-inflammatory and antioxidative properties (Guo et al., 2016). Concerning the anti-inflammatory effects, numerous experiments have demonstrated that *B. subtilis* could alleviate the negative effect of pathogens' challenge on immunity (Park et al., 2020; Fatholahi et al., 2021; Jiang et al., 2021; Xu et al., 2021). For instance, *B. subtilis* attenuated NE-challenge in broilers by decreasing inflammatory cytokines such as IL-6, IL-1β, IFN-γ, and TNF-α in infected birds (Wang et al., 2021b). Likewise, *B. subtilis* PB6 was reported to alleviate the immune-suppressive effect of NE in broiler chickens at similar efficacy to antibiotics (Liu et al., 2021).

Despite the overall comparable efficacy of *B. subtilis* with antibiotics at alleviating NE prevalence, our result suggested that *B. subtilis* could not completely replace antibiotics to control NE incidence, especially in a moderate to severe NE when high mortality and morbidity occur. The better efficacy of antibiotics compared to *B. subtilis* to lower mortality caused by NE was not fully elucidated in the studies involving NE-challenged broiler chickens. The strong bactericidal and bacteriostatic characteristics of antibiotics may facilitate better control against pathogens including *C. perfringens* and *Eimeria* species (Upmanyu and Malviya, 2020). In the studies included in this meta-analysis, several antibiotics were used as a current control measure of NE and coccidiosis including Zn bacitracin, salinomycin, bacitracin methylene disalicylate (**BMD**), narasin, flavomycin (Park et al., 2020; Keerqin et al., 2021; Liu et al., 2021; Oladokun et al., 2021; Wang et al., 2021b; Yang et al., 2021; Zhang et al., 2021b). Most of those studies reported nonsignificant differences in mortality compared to *B. subtilis* with few reporting numerically lower mortality rates. Additionally, as shown by the lesion score data, *B. subtilis* was shown to markedly ameliorate the severity of NE as indicated by the lower lesion score of challenged birds. This result also indicated that the ameliorative effects of *B. subtilis* may depend on a variety of factors especially the dosage and the severity of the NE or coccidiosis challenge. In subclinical conditions with low to moderate events of NE, *B. subtilis* might offer a better alternative for sustainable management practices on farms. This can also be a promising approach to reducing AMR potential from the use of excessive antibiotics. This is especially true because the administration of antibiotics as NE control agents or coccidiostats has been associated with the increasing number of reports of the development of drug-resistant strains of *C. perfringens* in broilers (Diarra et al., 2007; Gholamiandehkordi et al., 2009; Parker et al., 2021).

Furthermore, our analysis revealed that the strain of birds did not affect the outcomes while rearing phases, sex, and country of origin had a significant influence on the measured outcomes. The discrepancies due to these factors were anticipated, as NE incidence mostly occurred during grower or finisher periods rather than starter (Abd El-Hack et al., 2022; Pietruska et al., 2023) and male and female broilers have a different immunological and physiological responses against environmental challenges (Bozkurt et al., 2012; Zhang et al., 2021a). Countries' effects might explain different experimental designs rather than environmental conditions because all experiments were conducted in controlled-environmental settings, although the magnitude of the effects was consistent across studies.

Overall, while this meta-analysis confirmed that *B. subtilis* appears to be a promising alternative for the control of NE and coccidiosis in broiler chickens, more research is needed to fully compare the effectiveness of *B. subtilis* to traditional antibiotics. However, there is still a long way to go to address the issue of AMR in poultry production, and continued efforts and research are needed to develop and implement effective solutions. In particular, investigation of rotational or combinational use of *B. subtilis* and antibiotics as medication or preventive programs should be considered to suppress AMR in broiler chickens. In addition, the different doses of *C. perfringens* or *Eimeria* species used to infect broilers across studies might also contribute to effect size which was not accounted for in this study, due to the limited sample size and large variability of the doses. One might also be interested in comparing the efficacy of other feed additives. The present meta-analysis does not cover other feed additives that can be potentially used to ameliorate NE or coccidiosis, as recently reviewed (Adhikari et al., 2020). Therefore, it is an opportunity to perform a more comprehensive meta-analysis covering other feed additives, for example, using network meta-analysis or other models.

## CONCLUSIONS AND FUTURE PERSPECTIVE

The present meta-analysis highlighted important evidence that the use of *B. subtilis* as direct-fed probiotics is beneficial, either as a growth enhancer or as a protective management strategy to reduce the deteriorative effects of *C. perfringens* or *Eimeria* infections-induced NE disease. It was demonstrated that *B. subtilis* was on par with antibiotics to improve the disrupted performance of broiler chickens under NE-challenged conditions. However, antibiotics were superior to the lower mortality rate of NE-challenged broiler chickens. This evidence suggests that *B. subtilis* is effective and can be an ideal future probiotic to replace in-feed antibiotics to control the prevalence of NE or coccidiosis, although antibiotics administration may be required for severe infectious conditions.

Despite positive trends in probiotic product developments and adoption, several limitations warrant future work to improve the efficacy of *B. subtilis*-based probiotics in order to fully replace in-feed antibiotics especially for disease prevention and medication. Such limitations include cost-benefit ratio compared to antibiotics, low-cost downstream or industrial production scale, shelf life and storage, and stability and efficacy of *B. subtilis* strains in various environmental and farm conditions. The rapid development of genomic and bioinformatic tools can be used for the discovery study and to enhance the strains’ quality of *B. subtilis*, in addition to the development of effective and low-cost growth media, carriers, and manufacturing processes. Finally, supportive regulations need to be established to gradually reduce the use of antibiotics and replace them with natural-based additives.
